# Comparative analysis of the composition and function of fecal‐gut bacteria in captive juvenile *Crocodylus siamensis* between healthy and anorexic individuals

**DOI:** 10.1002/mbo3.929

**Published:** 2019-09-04

**Authors:** Mao Lin, Chenxi Zeng, Zhongqin Li, Ying Ma, Xueqing Jia

**Affiliations:** ^1^ Engineering‐Technology Research Center for Fishery Medicine, Fisheries College Jimei University Xiamen China; ^2^ Key Laboratory of Healthy Mariculture for the East China Sea Ministry of Agriculture Xiamen China

**Keywords:** anorexia, bacterial diversity, *Crocodylus siamensis*, gut microbes, microbial function

## Abstract

The Siamese crocodile (*Crocodylus siamensis*) is a freshwater, endangered crocodile with high economic value in the farming industry. Gut microflora plays an essential role in host physiological activity, and it contributes significantly to both the health and diseased states of animals. However, thus far, no study has focused on the correlation between diseases and intestinal bacterial communities in crocodilians. Here, we first compared the composition and function of gut microbial communities in captive juvenile *C. siamensis* suffering from anorexia and healthy crocodile controls using deep amplicon sequencing. The gut microbial diversity of anorexic crocodiles was much lower than the healthy individuals. Obvious changes in gut microbial composition were observed between sick and healthy crocodiles, except for *Cetobacterium somerae* of phylum Fusobacteria. In particular, the abundance of *Bacteroides luti*, *Clostridium disporicum*, *Plesiomonas shigelloides*, and *Odoribacter* sp. in the gut flora of healthy crocodiles was distinctly higher than the diseased group. Conversely, the species *Edwardsiella tarda* was overrepresented in the gut of anorexic crocodiles compared to the healthy group. Furthermore, in anorexic crocodiles, the predicted microbial functions that were related to amino acid metabolism, biosynthesis of other secondary metabolites, nucleotide metabolism, replication and repair, and translation were significantly reduced, while signal transduction was significantly enriched. These findings of the present study provide a reference to enrich the field of gut microorganism studies in crocodilians and suggest that alterations in the composition and function of gut bacteria in *C. siamensis* juveniles may be associated with anorexia in crocodiles.

## Graphical abstract

In this study, we demonstrate for the first time, the diversity of intestinal bacterial communities in captive juvenile *Crocodylus siamensis* suffering from anorexia and healthy crocodile controls using deep amplicon sequencing. Our findings provide a reference to enrich the field of gut microorganism studies in crocodilians and suggest that alterations in the composition and function of gut bacteria in *C. siamensis* may be associated with anorexia in crocodilians

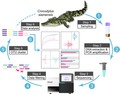

## 1 INTRODUCTION

The Siamese crocodile (*Crocodylus siamensis*) is an endangered freshwater crocodilian that is native to most countries in Southeast Asia, including Cambodia, Laos, Indonesia, Thailand, and Vietnam (Bezuijen et al., 2013). As far back as the mid‐1980s, wild crocodiles were exported from Cambodia to China as farm animals (Guo et al., 2018). In China, successful breeding of *C. siamensis* only occurs in a few southern provinces, such as Fujian and Hainan, because wild crocodiles have particular climate and temperature requirements for a suitable habitat (Guo et al., 2018). In addition to the requirements of the environmental conditions, bacterial diseases have also severely restricted the development of the crocodile farming industry (Camus & Hawke, 2002; Kim, Lee, & Kwak, 2016; Roh et al., 2011). The captive Siamese crocodile is universally acknowledged as “soft gold in aquatics” because it has significant economic benefits. Their skin is used in the leather industry, and their blood has potential effects in antibiotic therapy (Leelawongtawon, Siruntawineti, Chaeychomsri, & Sattaponpan, 2010). Their oil is used for medical treatment, and crocodiles are beneficial to the tourism industry (Li et al., 2012; Ryan, 1998).

The intestinal tract is an indispensable digestive organ that plays a key role in the defense of animals' immune system, and it is where considerable amounts of microbial flora colonize (Eckburg, 2005). Normally, the gut microflora is interdependent and interactive, which maintains the homeostasis of the internal environment, and it greatly influences the physiological activities of the host (Hooper, 2001). The composition and structure of the vertebrates' gut microbial communities are influenced by multiple factors (such as diet and environmental conditions) that also contribute to disease (Feng, Chen, & Wang, 2018; Scott, Gratz, Sheridan, Flint, & Duncan, 2013; Sharpton, 2018).

Prior studies on the gut bacterial communities of nonmammalian vertebrates have performed on birds, fish, amphibians, and reptiles (Colston & Jackson, 2016; Waite & Taylor, 2015). However, so far, the study on crocodilian's gut microbiome is still scarce, there was only one crocodilian species, American alligator (*Alligator mississippiensis*), which has been reported (Keenan, Engel, & Elsey, 2013). In those alligators, Fusobacteria was a unique and core flora of the gut microbiome, which is distinguished from other reptiles' gut microbiome (mainly consisted of Firmicutes and Bacteroidetes; Colston & Jackson, 2016; Keenan & Elsey, 2015). To expand the understanding of gut microflora of crocodilians, here we perform the 16S rRNA gene amplicon sequencing to compare the diversity of gut bacteria in healthy and anorexic Siamese crocodiles. The data observed from this work will elucidate the basic composition and function of the gut microbial communities in farmed crocodiles, and it will identify key bacteria that may influence the healthy growth of crocodilian.

## 2 MATERIALS AND METHODS

### 2.1 Sample collection

In May 2016, a sudden disease occurred in captive *Crocodylus siamensis* juveniles (1‐year‐old), which was observed at the Xiamen Lonsun crocodile zoo in Fujian Province. The clinical symptom of the sick Siamese crocodiles was anorexia (apparent decrease in daily feeding activity) with no trauma. The experimental crocodiles (0.65–0.78 m, 1.04–1.37 kg) were divided into two groups: the healthy group (labeled as H, *n* = 3) and the diseased group (labeled as D, *n* = 3). Six crocodiles were fed the same diet and reared in individual feeding areas before sampling. Cloacal swabs were used for sampling the crocodile gut flora which were an acceptable source for nondestructive sampling the reptiles' intestinal microbiota (Colston, Noonan, & Jackson, 2015; Jiang et al., 2017; Johnston, Porter, Scott, Rhodes, & Webster, 2010). The cloacal samples of the H group were labeled as H1–H3, while the cloacal samples of the D group were labeled as D1–D3. Specimens were stored in liquid nitrogen and immediately transported to the laboratory for DNA extraction.

### 2.2 DNA extraction

The total bacterial genomic DNA was extracted directly from each frozen sample (220 mg) using the PowerFecal^®^ DNA Isolation Kit (Qiagen), following the manufacturer's protocol. The quality and integrity of each DNA extraction was determined using 1% agarose gel electrophoresis before deep sequencing.

### 2.3 Deep amplicon sequencing

The genomic DNA was sequenced by the Majorbio Bio‐technology Company using the Illumina MiSeq PE300 platform (Illumina). PCR primers 338F (5′‐ACTCCTACGGGAGGCAGCAG‐3′) and 806R (5′‐GGACTACHVGGGTWTCTAAT‐3′) with dual barcode sequences were used to amplify the V3–V4 region of the 16S rRNA gene for all DNA samples. Each 20 μl of PCR mixture included 5× FastPfu buffer (4 μl), FastPfu Polymerase (0.4 μl), 2.5 mM dNTPs (2 μl), 5 μM forward primer (0.8 μl), 5 μM reverse primer (0.8 μl), and template DNA (10 ng). The PCR protocol was amplified using the conditions as following: 95°C for 3 min (initial denaturation); 25 cycles of 95°C for 30 s (denaturation), 55°C for 30 s (annealing), 72°C for 45 s (elongation), and 72°C for 10 min (final elongation). Then, the PCR products were detected by gel electrophoresis using 2% agarose and Tris–acetate–EDTA buffer, and finally, the amplicons (reads with an average length of 468 bp) were used for paired‐end sequencing analysis.

### 2.4 Bioinformatic and statistical analysis

**Figure 1:**
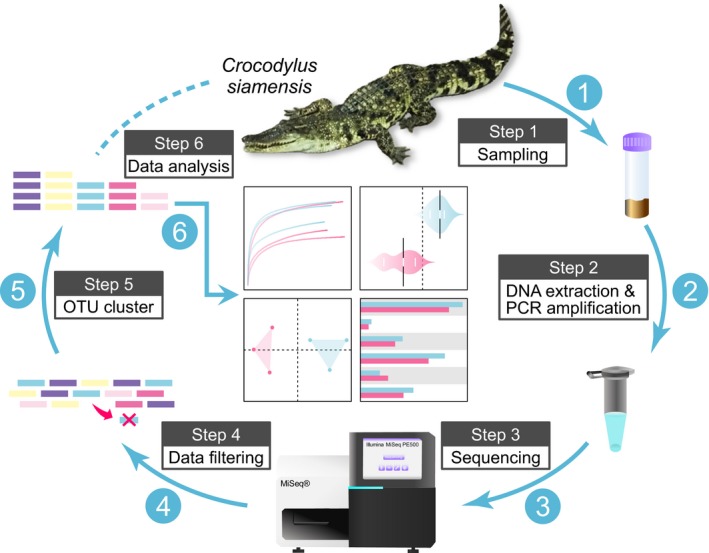
Brief description of the analysis approach for the gut microbial diversity of healthy and anorexic captive *Crocodylus siamensis*

Raw amplicon sequences obtained by deep sequencing were demultiplexed, quality‐filtered, and analyzed by using the software Mothur v1.35.1 (Schloss et al., 2009). All unqualified sequences, such as joint pollution, primer mismatches, low complexity, incorrect barcodes, and ambiguous bases, were discarded. Reads with a Q (base quality score) <20 and tags with less than 80% of the total base number were also removed. After the filtering and trimming procedures, all unique tags observed from each group were clustered into operational taxonomic units (OTUs) with a 3% distance level using Usearch v7.0 software (Edgar, 2010). Finally, all OTUs were classified taxonomically through the Ribosomal Database Project (RDP) Classifier, which is based on Naive Bayesian, with an 80% confidence threshold (Wang, Garrity, Tiedje, & Cole, 2007). As a result, the ACE, Chao1, Shannon, and Simpson indexes were calculated using Mothur v.1.35.1 software. The principal coordinates analysis (PCoA, weighted UniFrac distances) and bacterial taxa analysis were calculated and drawn in R v3.5.2 software. The alpha diversity indexes and relative abundance of gut microbial communities (phylum and genus level) that were identified from healthy (*n* = 3) and diseased (*n* = 3) groups were comparatively analyzed by Student's *t* test, and *p* < .05 was considered significant. Linear discriminant analysis (LDA) effect size (LEfSe) was employed to determine the key contributors of gut bacteria in healthy and anorexic crocodiles, and the LDA score threshold was 3.5 (Segata et al., 2011). Furthermore, PICRUSt analysis via the Kyoto Encyclopedia of Gene and Genomes (KEGG) database was used to predict functional profiles of gut bacteriome in healthy and diseased crocodiles (Langille et al., 2013). STAMP v2.1.3 software was used to statistically analyze the gene functions using Student's *t* test with Bonferroni correction (Parks, Tyson, Hugenholtz, & Beiko, 2014). A *q*‐value (adjusted *p*) < .05 with an effect size >0.2 was considered significant. A brief description of the total bioinformatic analyses of crocodile gut genomic DNA is shown in Figure 1.

## 3 RESULTS

### 3.1 Sequence survey

A total of 258,748 valid reads were gathered from cloacal samples from 6 individual crocodiles. The number of effective unique tags ranged from 38,125 to 48,216 per sample, resulting in 168 OTUs with same sequence similarity values of 97% (Table 1). The number of OTUs obtained from each sample ranged from 51 to 88. In this study, 99.98%–99.99% coverage of species was obtained in all samples, which demonstrates that the majority of the bacterial phylotypes present in the specimens were identified.

**Table 1:** Summary of species richness estimators, including observed sequence reads, OTUs, estimated OTU richness (ACE and Chao1), diversity index (Shannon and Simpson), and estimated sample coverage between different cloacal samples

Sample	Reads	OTUs	ACE	Chao1	Shannon*	Simpson*	Coverage
H1	43,776	88	92.22	91.50	2.41	0.16	99.98%
H2	41,681	84	87.91	85.88	2.52	0.13	99.99%
H3	38,125	66	75.11	72.43	2.16	0.20	99.98%
D1	48,216	51	52.29	52.20	1.01	0.44	99.99%
D2	39,584	58	63.33	60.15	1.49	0.33	99.98%
D3	47,366	88	93.58	93.14	1.79	0.28	99.98%

Note

H1 to H3 represent the healthy crocodile cloacal samples. D1 to D3 represent the anorexic crocodile cloacal samples. OTUs clustered at 97% sequence identity. * indicates a significant difference between the healthy group (contained H1, H2, and H3) and the diseased group (contained D1, D2, and D3), as determined by Student's *t* test. *p* < .05 was considered significant.

### 3.2 Alpha and beta diversity

**Figure 2:**
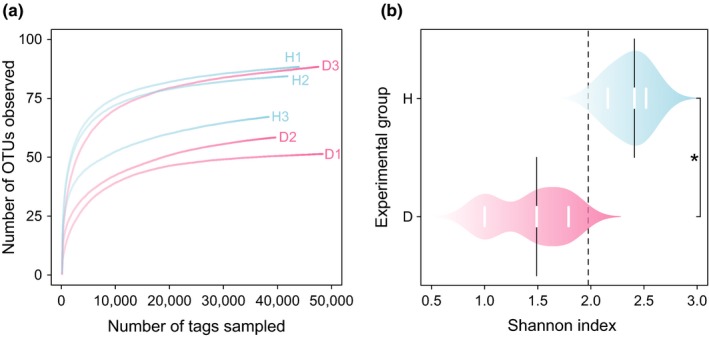
Alpha diversity of the gut microflora of captive Siamese crocodiles. (a) Rarefaction curve sequences show the species richness in the healthy group (H1, H2, and H3) and the diseased group (D1, D2, and D3) at the 3% distance cutoff. (b) The Shannon indexes of the cloacal samples from 6 individual crocodiles. H (*n* = 3) indicates the healthy group. D (*n* = 3) indicates the diseased group. * indicates a significant difference between the H group and the D group, as determined by Student's *t* test. *p* < .05 was considered significant

**Figure 3:**
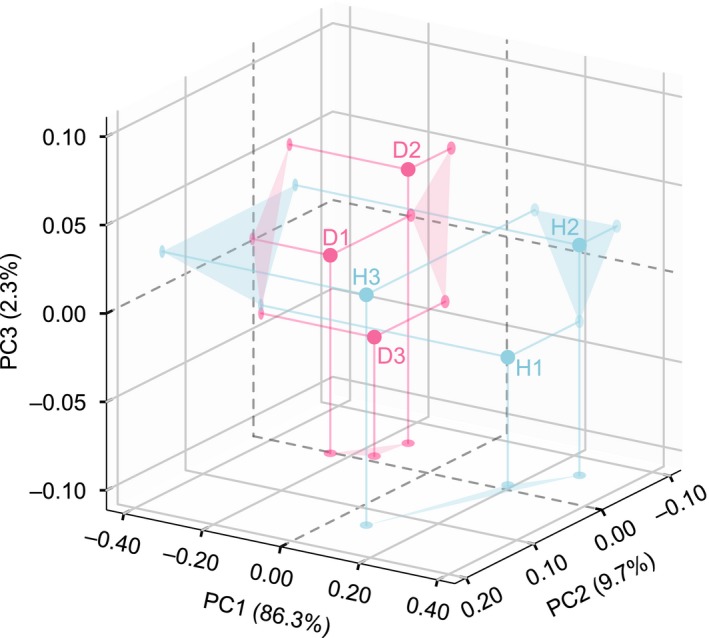
Principal coordinate analysis (PCoA) of differences in gut microbial communities based on the weighted UniFrac distances observed from 6 individual crocodiles. H indicates the healthy crocodile group (containing H1, H2, and H3). D indicates the diseased crocodile group (containing D1, D2, and D3)

The rarefaction curve of the D group quickly reached the saturation plateau under 97% similarity values, which indicates lower species richness compared to the H group (Figure 2a). The Shannon indexes of the gut communities (Table 1; Figure 2b) in the H group were significantly higher (*p* = .021) than in the D group, and the Simpson indexes (Table 1) were significantly lower (*p* = .022) than the D group. These results indicate that the H group had richer microbial diversity than the D group. The PCoA score plot (Figure 3) revealed that the PC1, PC2, and PC3 axes included almost all variations (98.3%) of principal components found among the cloacal samples from 6 individual crocodiles. However, the H group samples were separated from the D group samples along the vast major component PC1 axis, which accounted for 86.3% of total variations.

### 3.3 Taxonomic composition and comparison

**Figure 4:**
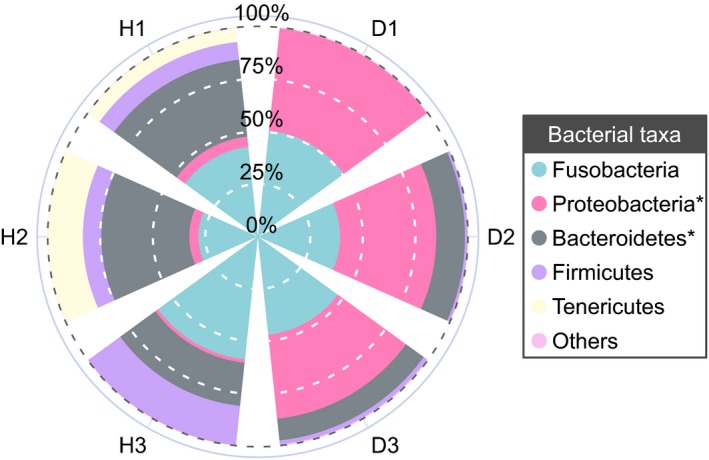
Relative abundance of gut bacterial composition of 6 individual crocodiles, organized at the phylum level. H1, H2, and H3 represent the healthy group; D1, D2, and D3 represent the diseased group. Genera with an observed relative abundance less than 1% and unclassified bacteria in both groups were assigned as “Others.” * indicates a significant difference between the healthy group and the diseased group, as determined by Student's *t* test. *p* < .05 was considered significant

At the phylum level (Figure 4), the core microbes in the H (H1, H2, and H3) and D (D1, D2, and D3) libraries were Fusobacteria (H: 43.30%; D: 45.57%, *p* = .82), Bacteroidetes (H: 33.14%; D: 8.06%, *p* = .03, significantly enriched in the H group), Firmicutes (H: 12.03%; D: 1.10%, *p* = .82), Tenericutes (H: 7.79%; D: <0.01%, *p* = .18), and Proteobacteria (H: 3.63%; D: 44.96%, *p* < .001, significantly enriched in the D group).

**Figure 5:**
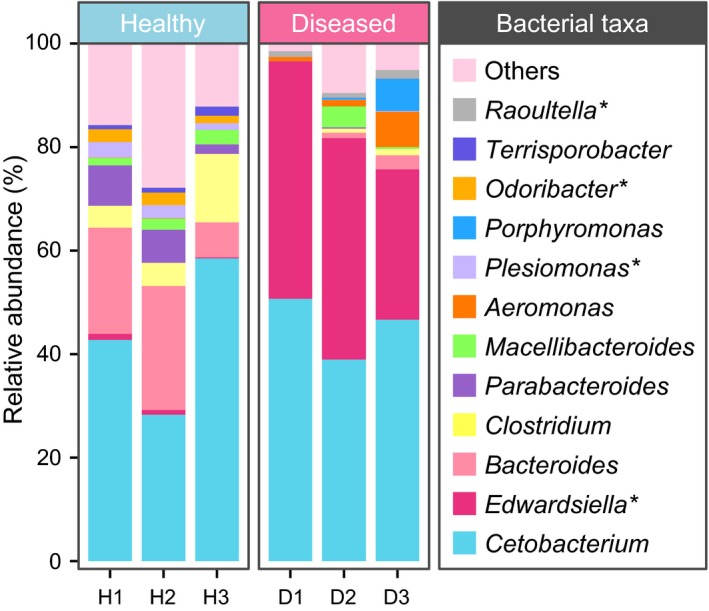
Relative abundance and composition of gut bacteria from 6 individual crocodiles, organized at the genus level. H1, H2, and H3 indicate the healthy group; D1, D2, and D3 indicate the diseased group. Genera with an observed relative abundance less than 1%, and unclassified bacteria in both groups were assigned as “Others”. * indicates a significant difference between the healthy group and the diseased group, as determined by Student's *t* test. *p* < .05 was considered significant

At the genus level (Figure 5), the H libraries displayed a distinct structure of bacterial composition (mean relative abundance >1%) from the D libraries, except for *Cetobacterium* (*p* = .82). This shared genus was also the most dominant genus identified in all samples; it accounted for 43.15% in the H group and 45.40% in the D group. The common gut microbial communities presented in both the H and D groups also included *Bacteroides* (H: 17.10%; D: 1.26%, *p* = .09) and *Macellibacteroides* (H: 2.14%; D: 1.45%, *p* = .64). Moreover, the genera *Clostridium* (7.33%, *p* = .23), *Parabacteroides* (5.33%, *p* = .10), *Plesiomonas* (2.29%, *p* = .05, significantly enriched), *Odoribacter* (2.09%, *p* = .03, significantly enriched), and *Terrisporobacter* (1.17%, *p* = .07) were the dominant bacteria in the H group. The major components of the D group were *Edwardsiella* (39.28%, *p* = .02, significantly enriched), *Aeromonas* (3.01%, *p* = .27), *Porphyromonas* (2.19%, *p* = .39), and *Raoultella* (1.18%, *p* = .04, significantly enriched).

Specifically, all sequences recognized in the two sample groups that were within the genera *Cetobacterium*, *Edwardsiella*, *Aeromonas*, *Plesiomonas*, *Terrisporobacter*, and *Raoultella* belonged to *Cetobacterium somerae*, *Edwardsiella tarda*, *Aeromonas hydrophila*, *Plesiomonas shigelloides*, *Terrisporobacter petrolearius*, and *Raoultella planticola*, respectively. Furthermore, *Bacteroides luti*, *Clostridium disporicum*, and *Porphyromonas pogonae* were the major sequence contributors (69.82%, 39.71%, and 99.7%, respectively) from the genera *Bacteroides*, *Clostridium*, and *Porphyromonas*, respectively.

### 3.4 Significant alterations of the gut microbial community in healthy and diseased Siamese crocodiles

**Figure 6:**
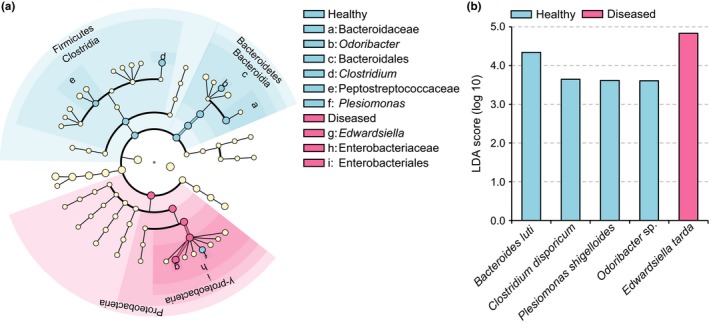
The linear discriminant analysis (LDA) effect size (LEfSe) analysis that shows the significant differences in gut flora between healthy and diseased groups. (a) Cladogram plot demonstrating the significant gut bacteria in both healthy and diseased groups. (b) Highly significant bacterial species with an LDA score >3.5

In this study, LEfSe analysis was employed to identify any key contributors that have a statistically significant role in the H and D groups. These data were calculated and analyzed by the nonparametric factorial Kruskal–Wallis test and pairwise Wilcoxon test with the same *p* value of .05. An LDA score value > 3.5 was considered to have reached statistical significance. The cladogram plot (Figure 6a) demonstrated that the two groups could be separated at the phylum level of the significant bacteria. It indicated that the H group contained all Firmicutes and Bacteroidetes, whereas the D group contained mostly Proteobacteria. Compared to the D group, reads from the H group indicated that the species *B. luti* (LDA score = 4.34), *C. disporicum* (LDA score = 3.65), *P. shigelloides* (LDA score = 3.62), and *Odoribacter* sp. (LDA score = 3.61) may have a highly significant effect on the healthy growth of crocodiles based on their LDA score (LDA score > 3.5). In addition, the species *E. tarda* (LDA score = 4.83) was significantly enriched in the D group, and it may have a negative effect on growth (Figure 6b).

### 3.5 Comparison of the functional profiles of gut flora from healthy and diseased Siamese crocodiles

**Figure 7:**
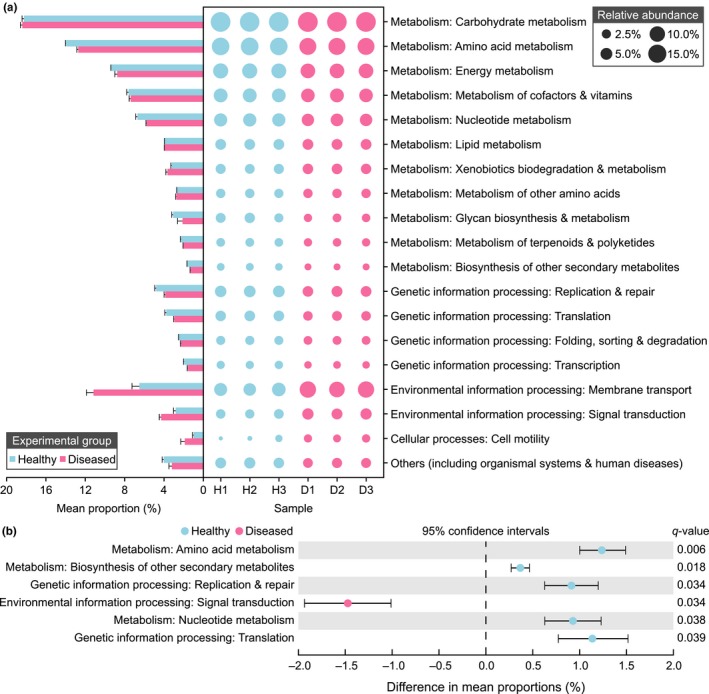
Predicted gut functional composition and the differences between the healthy and diseased groups. These were predicted by PICRUSt against the KEGG pathway database (at level 1 and level 2). (a) Functional families of healthy (H1, H2, and H3) and diseased (D1, D2, and D3) cloacal samples. (b) Extended error bar plot showing the significant differences in gene functions against KEGG database pathways (at level 2). The *q*‐value (adjusted *p*) was tested by Student's *t* test and multiple‐corrected using the Bonferroni method. A *q*‐value <.05 and effect size >.2 were considered significant

The present study used PICRUSt analysis to predict the major gene functions to determine the functional profiles of the gut microflora in the H and D groups. The identified genes that were predicted in all specimens, which were primarily involved in the KEGG level 1 pathways of metabolism (H: 72.68%; D: 68.79%), genetic information processing (H: 13.00%; D: 10.68%), environmental information processing (H: 9.32%; D: 15.45%), and cellular processes (H: 0.98%; D: 1.89%). These were further assigned as dominant predicted genes of 18 functional categories of KEGG level 2 pathways (Figure 7a). When compared to the H group (Figure 7b), several functional pathways (at level 2) of metabolism and genetic information processing were significantly reduced (*q*‐value <.05, effect size >.2). The pathways of metabolism included amino acid metabolism, biosynthesis of other secondary metabolites, and nucleotide metabolism. The pathways of genetic information processing included replication, repair, and translation. Moreover, the environmental information processing function that is related to signal transduction was significantly enriched (*q*‐value <.05, effect size >.2) in the D group. This was revealed by STAMP analysis using Student's *t* test coupled with Bonferroni correction.

## 4 DISCUSSION

Gut microbiota is generally recognized as an indivisible “organ” of the host that is closely related to various diseases in animals (Dou et al., 2017; Roy et al., 2013; Li et al., 2017; Nicholson et al., 2012; Stanley, Hughes, & Moore, 2014). In the entire dataset, the gut microbes (mean relative abundance >10%) identified in healthy crocodiles were dominated by the phyla Fusobacteria, which was followed by Bacteroidetes and Firmicutes. According to previous studies that sequenced 16S rRNA genes, Bacteroidetes and Firmicutes were found to be the core gut communities of most amphibians and reptiles (Bletz et al., 2016; Colston et al., 2015; Jiang et al., 2017; Kohl et al., 2016). However, the Fusobacteria were dominated in the gut microbiota of freshwater American alligators, which is consistent with the current study (Keenan et al., 2013). As a result, there was a substantial alteration in the gut microbial composition between anorexic crocodiles and healthy crocodiles. An exception was the species *C. somerae* because it was the most dominant bacterium identified in both the healthy and diseased groups. *C. somerae* was first isolated from human feces, and it has been universally identified in the gut of freshwater fish (Bledsoe, Peterson, Swanson, & Small, 2016; Larsen, Mohammed, & Arias, 2014; Lin et al., 2019). It has been found to produce vitamin B12 and acetic acid, which are beneficial for the host (Finegold et al., 2003; Tsuchiya, Sakata, & Sugita, 2008). However, *C. somerae* do not seem to be strongly associated with healthy Siamese crocodiles.

The proportion of Bacteroidetes and Firmicutes in the healthy group was higher than it was in the diseased group. Among the Bacteroidetes, *Bacteroides* was the most abundant genera in the healthy group, and the species *B. luti* was significantly enriched compared to the diseased group. *Bacteroides* sp. is a component in the gut flora of various vertebrates, including carps, cottonmouth snakes, and crocodile lizards, and *B. luti* was first isolated from methanogenic sludge (Colston et al., 2015; Hatamoto, Kaneshige, Nakamura, & Yamaguchi, 2014; Jiang et al., 2017; Li et al., 2015). However, the effect of *B. luti* on the host‐bacteria ecosystem is vastly underexplored. *Odoribacter* was also a core genus of the Bacteroidetes phylum observed in this study, and it demonstrated a positive correlation with crocodile health. This was closely related to the human gut microflora and may improve host metabolism (Kulagina et al., 2012; Lim et al., 2017). Firmicutes was the third‐most dominant phylum identified in healthy crocodile cloacal samples, which mainly consisted of the genus *Clostridium*. According to previous studies, most *Clostridium* sp. can produce a type of fatty acid called butyrate, which provides many benefits to the health of the host gut (Hamer et al., 2009; Pryde, Duncan, Hold, Stewart, & Flint, 2002). The *Clostridium* organisms observed in the healthy group had the highest bacterial diversity, which included 9 different kinds of *Clostridium* species (all mean relative abundance >0.1%). However, only *C. disporicum* reached statistical significance when compared to the diseased group. *C. disporicum* is an uncommon, fermentative, and anaerobic gut bacterium, and it is primarily found in mammal feces, such as rats and pigs (Horn, 1987; Su, Yao, Perez‐Gutierrez, Smidt, & Zhu, 2008). It has been found to be related to the degradation of complex organic macromolecules (Vilajeliu‐Pons et al., 2015). Moreover, we found that the rarely known *T. petrolearius* was also a major component in the gut of healthy crocodiles’ group. *T. petrolearius* was first isolated from oilfields, and this is the first report that suggests this microbe is associated with animal gut flora (Deng et al., 2015). Interestingly, the present study found that the human pathogen *P. shigelloides* in the phyla Proteobacteria was also significantly enriched in the cloacal samples of healthy crocodiles (Chen et al., 2013). This appears to be a normal component of the gut flora of aquatic animals (Johnston et al., 2010; Larsen et al., 2014; Lin et al., 2019; Silva, Brito, Farias, & Nicoli, 2005). In contrast, the intestinal microflora of diseased Siamese crocodiles was significantly enriched in *E. tarda,* which belongs to the phyla Proteobacteria. *E. tarda* is widely known as a zoonotic pathogen, and it is generally found in animals of an aquatic environment, such as bullfrogs, alligators, and eels (Johnston et al., 2010; Lin et al., 2019; Mauel, Miller, & Frazier, 2002). In addition, *E. tarda* can infect a broad range of hosts and cause various diseases, which most frequently present as gastroenteritis and septicemia (Leung, Siame, Tenkink, Noort, & Mok, 2012; Miyazawa et al., 2018; Wang et al., 2005).

Furthermore, the result of the functional prediction indicated that the diseased group had a reduction in pathways related to amino acid metabolism, biosynthesis of other secondary metabolites, nucleotide metabolism, replication and repair, and translation, and there was a significant enrichment in the signal transduction pathway in the diseased group. Metabolism is a basic requirement for maintaining the normal growth of hosts, and this is commonly related to the function of gut bacteria in animals, such as snakes, mice, birds, and goats (Mclaughlin, Cochran, & Dowd, 2015; Suzuki & Nachman, 2016; Wang, Jin, Xue, Wang, & Peng, 2019; Wang et al., 2018). Healthy crocodiles demonstrated distinctly higher rates for some metabolic pathways. This may be related to higher energy consumption, which is required to fulfill the normal growth of the host. When some of the diseased crocodiles suffered from anorexia, there was a significant decrease in several metabolic functions of the gut microbes which were compared to the healthy controls. Besides, some functional pathways of cellular processes (including replication, repair, and translation) were also significantly reduced in the gut of anorexic crocodiles. Based on the prior work, it has been shown that the signal transduction system contributed to antibiotic resistance, biofilm formation, environmental persistence, virulence protein, and pathogenicity in *E. tarda* (Lv et al., 2012). Therefore, a high abundance of *E. tarda* may be strongly associated with an increase in the signal transduction pathway, and this may contribute to the negative effect observed in sick crocodiles. Thus, we hypothesized that during the growth stage of the captive Siamese crocodiles, some juvenile individuals might have been infected with some pathogens such as *E. tarda*, and then abnormal alterations of composition and function of healthy crocodiles' gut bacteria might be related to the disease by causing anorexia.

In conclusion, the present study is the first to report the composition and function of the gut microflora of captive juvenile Siamese crocodiles in both healthy and diseased conditions. The presence of *B. luti*, *C. disporicum*, *P. shigelloides*, and *Odoribacter* sp. may have beneficial contributions to the healthy growth of crocodiles, but *E. tarda* may negatively influence the health of the host. Our findings revealed that alterations in the composition and function of the intestinal bacteria of sick and healthy crocodiles might be associated with anorexia. However, it is still unclear how gut microbes interact with each other, which should be explored in further research. Besides, future studies should seek to increase the sample size of the host in order to enhance the statistical power for detailed bioinformatic analyses.

## CONFLICT OF INTERESTS

None declared.

## AUTHOR CONTRIBUTIONS

Conceptualization: ML (lead) and CXZ (supporting); Data curation: CXZ; Formal analysis: CXZ (lead), ML (equal), ZQL (equal), YM (supporting); Funding acquisition: ML; Investigation: CXZ (lead), ZQL (equal), YM (equal), and XQJ (supporting); Methodology: CXZ (lead), ZQL (equal), YM (equal), and XQJ (supporting); Project administration: ML; Supervision: ML; Software: CXZ (lead) and ZQL (supporting); Validation: CXZ (lead), ZQL (equal), YM (equal); Visualization: CXZ; Writing**–**original draft: CXZ (equal) and ML (equal). All authors reviewed and edited the manuscript, and gave the final approval for publication.

## ETHICS STATEMENT

This study complied to the guidelines for the kindly care and use of experimental animals established by the Ministry of Science and Technology of the People's Republic of China (Approval No. 2006–398). Besides, the research protocol was reviewed and approved by the Animal Ethics Committee of Jimei University (Approval No. JMULAC201603). No hunting and destructive sampling involved.

## Data Availability

All raw sequencing data obtained in this study were deposited in the Sequence Read Archive of the National Center for Biotechnology Information (NCBI), and the SRA submission data are SRX4396839‐SRX4396844.
